# Trimetallic (Au_rod_-Pd_shell_-Pt_cluster_) Catalyst Used as Amperometric Hydrogen Peroxide Sensor

**DOI:** 10.3390/bios4040461

**Published:** 2014-11-19

**Authors:** Shou-I Cheng, John Rick, Chun-Jern Pan, Hung-Lung Chou, Wei-Nien Su, Kuan-Jung Chen, Chung-Chiun Liu, Yaw-Wen Yang, Chia-Hsin Wang, Bing-Joe Hwang

**Affiliations:** 1 Nanoelectrochemistry Laboratory, Department of Chemical Engineering, National Taiwan University of Science and Technology, Taipei 106, Taiwan; E-Mails: assoonas2009@yahoo.com.tw (S.-I.C.); nouveauvous@yahoo.com (J.R.); pom9106054@yahoo.com.tw (C.-J.P.); d9706002@mail.ntust.edu.tw (K.-J.C.); 2 Graduate Institute of Applied Science and Technology, National Taiwan University of Science and Technology, Taipei 106, Taiwan; E-Mails: a8406033@mail.ntust.edu.tw (H.-L.C.); wsu@mail.ntust.edu.tw (W.-N.S.); 3 Department of Chemical Engineering, Case Western Reserve University, Cleveland, OH 44106, USA; E-Mail: cxl9@case.edu; 4 National Synchrotron Radiation Research Center, Hsinchu 30076, Taiwan; E-Mails: yang@nsrrc.org.tw (Y.-W.Y.); wang.ch@nsrrc.org.tw (C.-H.W.)

**Keywords:** trimetallic catalyst, Au core-shell, hydrogen peroxide sensing, nanoparticles, nanorods

## Abstract

Bimetallic nanostructured core-shell structures are commonly used as catalysts in a wide variety of reactions. We surmised that the addition of an additional metal would potentially allow catalytic tailoring with the possibility of an increase in activity. Here a tri-metallic catalytic structure, consisting of clustered catalytic Pt on the surface of a Pd shell supported on a rod shaped Au core was fabricated. The significance of the additional metallic component is shown by comparative electrochemically active surface area (ECSA) analysis results for the trimetallic Au_rod_-Pd_shell_-Pt_cluster_, bimetallic Au_rod_-Pt_cluster_ and monometallic JM-Pt (used as a reference), which have respective ECSA values (cm^2^/mgPt) of 1883.0, 1371.7 and 879. The potential utility of the trimetallic catalysts was shown in a hydrogen peroxide sensing protocol, which showed the catalyst to have a sensitivity of 604 ìA/mMcm^2^ within a linear range of 0.0013–6.191 mM.

## 1. Introduction

Catalysts incorporating metals with high catalytic ability, typically palladium and platinum, incur cost-penalties due to the scarcity of the active metal component; thus, structures that efficiently employ a minimal amount of material, while retaining high catalytic efficiency, are highly sought after. Core-shell structured catalysts, formulated to contain only small amounts of palladium and platinum, have been used in a range of catalytic applications [1].

The design history of catalytic materials shows a progression from simple mono-metallic catalysts, especially Pt, Pd and nano-dimensioned Au, to bimetallic structures: The addition of the second metal can not only help tailor sought after properties in terms of the particle’s size, shape, and surface morphology, but can also heighten catalytic efficiency by the transfer of electron density from the underlying support metal into the surface catalytic architecture. In addition to the presence of a second metal the importance of catalyst-support interactions has also recently come to prominence [2,3,4,5,6].

The introduction of a third metal into the system should give the possibility of further tailoring the catalyst’s performance. Some challenges and the outcome of interesting recent investigations are very briefly given below. Tompos *et al*., 2007 [7], have used a combinatorial library design approach to synthesise of trimetallic Pt-Pd-Au/CeO_2_ catalysts for total methane oxidation. It was shown that the addition of Pt and Au to palladium promoted the methane oxidation activity, as well as improved the long-term stability compared to the monometallic Pd catalyst. The authors also attribute enhanced activity to the formation of new active sites that adsorb methane more strongly than in the monometallic Pd catalyst. Yu *et al.*, 2013 [8], exploited a shape recovery phenomenon in Pt−Ni bimetallic nanocrystals attributable to defect effects. An intrinsic defect-dominated growth mechanism was shown to allow the site-selective nucleation of a third metal around the defects to design trimetallic Pt_3_Ni@M core-shell structures (M = Au, Ag, Cu, Rh). Fang *et al.*, 2011 [1], designed Au-core Pd-shell Pt-cluster nanoparticles for enhanced electrocatalytic activity and showed using extremely small amounts of Pt and Pd unusually high activity for electrooxidation of formic acid in fuel cells. The optimized structures had only two atomic layers of Pd and a half-monolayer equivalent of Pt; interesting, it was found that further increasing the loading of Pd or Pt reduced catalytic activity, suggesting the presence of a synergistic effect among the three nano-structural components.

In this study a tri-metallic structure was sequentially fabricated with the initial stage being the deposition of Pd on Au rods to give a Au_rod_-Pd_shell_ support for the later deposition of Pt: the ability to exploit high surface area to mass ratios of precious metals by using thin coating confers both enhanced activity/selectivity and considerable materials cost savings [4,5,9].

A practical evaluation of our trimetallic catalyst’s properties, to enable comparison with those of Au_rod_-Pt_cluster,_ Pt and catalysts [4,5,10,11,12], was made by comparative evaluation for hydrogen peroxide (H_2_O_2_) sensing. The accurate and reliable sensing of H_2_O_2_ is pivotal to a wide range of industrial, healthcare, and, more recently, anti-terrorism applications; some diverse examples being the bulk-scale bleaching of paper, aseptic packaging, bio-imaging, and the determination of clinically important analytes, such as glucose and uric acid. Despite its scientific and technological importance, the direct detection of hydrogen peroxide, resulting for example from the breakdown of glucose or urea by an oxidase enzyme, remains problematic, due to the absence of any UV absorbance or fluorescence activity. Thus, new approaches leading to the development of robust and sensitive H_2_O_2_ sensors remain an ongoing quest.

## 2. Experimental Section 

### 2.1. Apparatus and Reagents

The supporting electrolyte, used for peroxide sensing, was phosphate buffer solution (PBS) containing 0.10 M Na_2_HPO_4_–NaH_2_PO_4_ + 0.10 M KCl (pH 7.4), all other chemicals were of analytical grade and used without further purification. Solutions were prepared and diluted using ultra-pure water (Millipore Milli Q system). Electrochemical experiments were performed using an Autolab PGSTAT302N electrochemical analyzer (AUTOLAB, USA) with a conventional three electrode cell. The working electrode was a glassy carbon electrode (GCE, diameter 5 mm). A sliver/sliver chloride (Ag/AgCl) and a platinum wire electrode were used as the reference and counter electrodes, respectively.

Powder X-ray diffraction (XRD) patterns were obtained on a diffractometer (RigakuDmax-B, Japan) using a Cu K_α_ source operating at 40 kV and 100 mA (scanning rate of 0.05 degs^−1^ for 2θ values between 20° and 90°). Transmission electron microscopy (TEM) examination was performed on a Philips Tecnai F20G2 FEI-TEM microscope operating with an accelerating voltage of 200 keV. The EDX measurements were performed with a JSM 6500 EDX analyzer. Specimens were prepared by ultrasonically suspending the catalyst powders in ethanol, applying the specimen to a copper grid and drying in air. Scanning electron microscopy (SEM) images were obtained at 5.0 keV on a Hitachi S-240 JOEL JSM-6500F field emission SEM. X-ray spectra were recorded at the National Synchrotron Radiation Research Center (NSRRC) of Taiwan, Beam Line 01C1, following the procedure described in detail in our previous publications [13,14].

The synthesis method employed a sequential strategy (see below). Initially, Au nanorods were prepared using existing methods. These were used as a platform for the deposition of a Pd thin-shell, which in turn served as the support for the Pt clusters.

### 2.2. Synthesis of Au Nanorods

Our synthesis is based on seed-mediated growth in aqueous solutions, which has been used widely for the preparation of Au nanostructures [15]. Briefly, the seed solution was prepared by rapidly injecting NaBH_4_ (0.01 M, 0.6 mL) into a mixture of HAuCl_4_ (64.5 uL 38.76 mM) and CTAB (3.75 mL, 0.20 M) and kept in a water bath, maintained at 25 °C, prior to use. To grow Au nanoparticles, a growth solution was prepared, comprising CTAB (0.2 M, 18.4 mL), HAuCl_4_·3H_2_O (38.76 mM, 0.474 mL), and AgNO_3 _(0.368 mL, 0.01 M). To this solution was added ascorbic acid (0.2 M, 0.147 mL), then seed solution diluted 5 times with CTAB (0.1 M) to give a final volume of 100 μL was added into growth solution and left undisturbed overnight. The resulting nanocrystals were washed twice in the same solvent by centrifugation prior to use.

### 2.3. Synthesis of Au_rod_-Pd Shell Structured Nanoparticles

In a typical synthesis the above aqueous Au_rod_ solution (0.6 mL) and 14.4 mM aqueous K_2_(PdCl_4_) were added to DI water (41 mL). To this solution, 200 mM CTAB solution (8 mL) was added, after which ascorbic acid (200 mM, 80 μL) was rapidly injected with vigorous stirring during a period of 1 min. The solution was then heated in an oil bath at approximately 26 ± 2 °C 24 h, after which the particles were separated by centrifugation (8000 rpm for 6 min) to remove excess reagents [16].

### 2.4. Preparation of Au Rod-Pd Shell-Pt Cluster

To prepare the final catalytic structure DI water (42 mL), Au@Pd nanoparticles (1826 μL), K_2_PtCl_4_ (23.18 mM, 21.5 μL), and CTAB (200 mM, 49.8 mL) were mixed. To this mixture ascorbic acid (200 mM, 298 μL) was quickly added (within approximately 10 s) by injection. The resulting solution was heated in an oil bath 75 ± 2 °C for 15 min prior to being cooled in water. Centrifugation (5000 rpm/20 min/2 times) was used to isolate the final product.

### 2.5. Preparation of H_2_O_2_ Sensing Electrode

A glassy carbon electrode was polished, using Al_2_O_3_ powder, to remove surface impurities. The as-prepared catalyst was dispersed in 2 mL ultrapure water under sonication. Catalyst slurry, 15 μL, (transferred by pipette) was dropped onto the electrode’s surface prior to oven drying.

### 2.6. Preparation of H_2_O_2_ Solution

H_2_O_2_ (4.280 mL, 11.68 M) was diluted with phosphate buffer solution (0.1 M, pH = 7.4) to a final volume of 50 mL to give H_2_O_2_ (1 M). The solution was stored at 4 °C prior to use.

### 2.7. H_2_O_2_ Sensing

Amperometric measurements were conducted to investigate H_2_O_2_ catalytic sensitivity. The applied potential was set to 0.4 V, with aliquots of H_2_O_2_, of known concentration, being added at 20 s intervals. A 5 s reaction time was allowed prior to measuring the variation in current response.

## 3. Results and Discussion

### 3.1. Characterization

**Figure 1:**
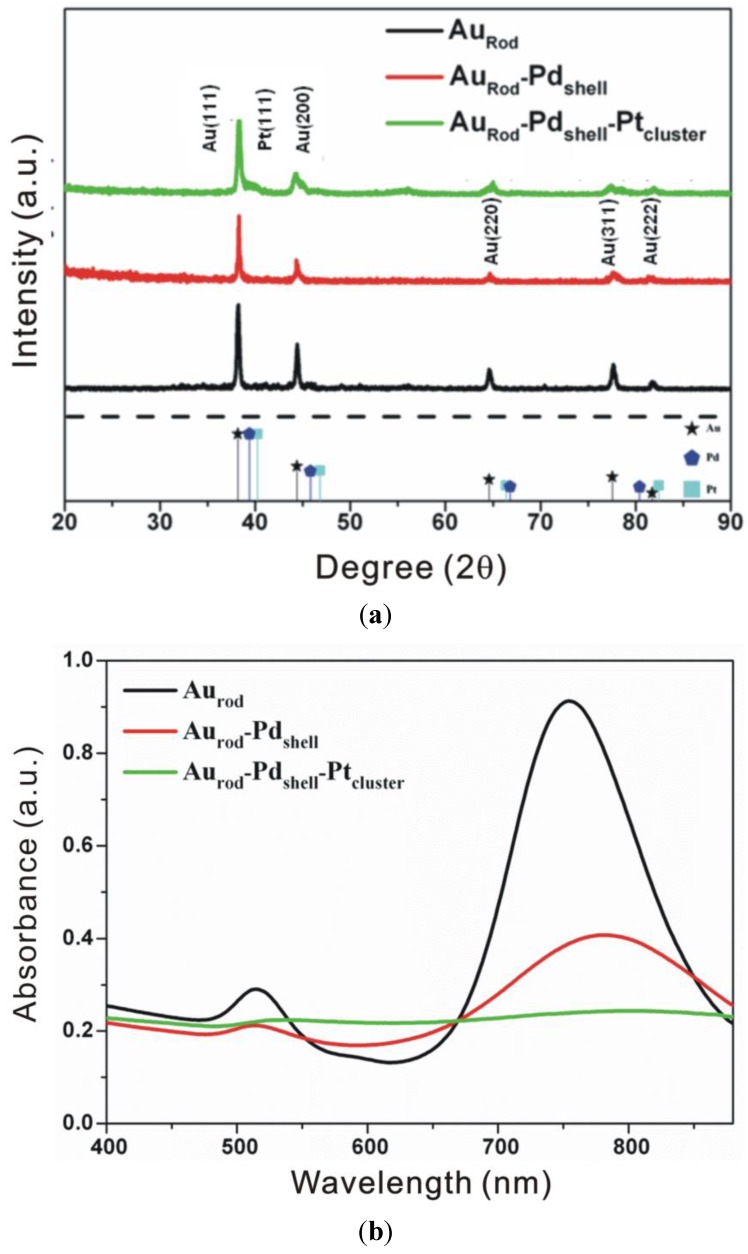
Material characterisation showing (**a**) XRD patterns and (**b**) UV spectra evolution as each additional added layer of the composite catalyst is added.

**Figure 2:**
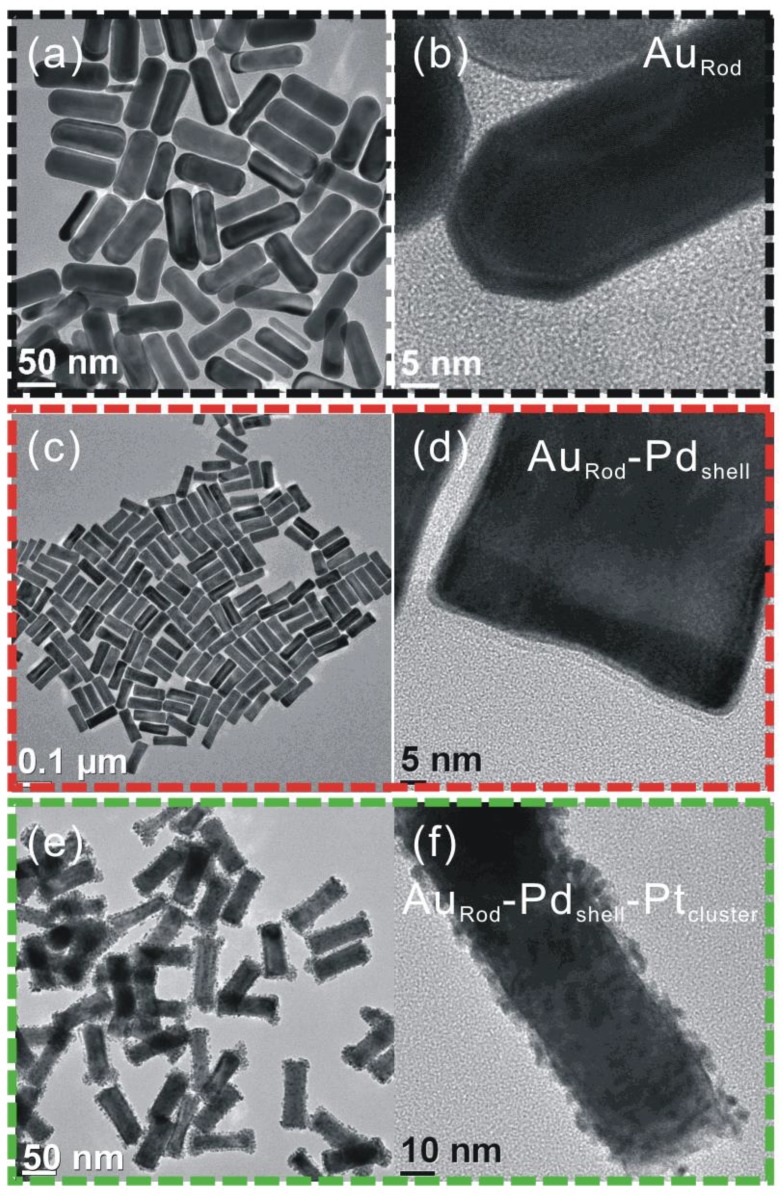
TEM images showing the stepwise formation of the catalyst: (**a**) Au rods; (**b**) Pd shell formed on the rods, and (**c**) after the formation of clustered Pt structures on the Pd shell.

The resulting nanoparticles were characterized by XRD, UV-vis, TEM, and XPS. Considering the XRD and UV spectra in conjunction with the TEM images clearly shows the formation of the composite catalyst, see Figure 1 and Figure 2.

From the TEM images, we can see that the core–shell nanostructures consist of Au rod-like cores (distorted octagonal structures Figure 2a,b) with a Pd coating (rectangular structures Figure 2c,d), upon which a coating comprising Pt clusters is deposited, Figure 2e,f.

The XRD peak positions of the core-shell nanoparticles carrying the Pt clusters are similar to those of the core-shelled nanoparticles because the particle size of the Pt clusters is too small to give a distinct diffraction peak; however, the presence of Pt is confirmed by a Pt(111) shoulder which is apparent on the Au(111) peak.

The UV/Vis spectrum of the Au_rods_ displays the surface plasmon resonance peaks (centered at ~510 and 750 nm) associated with the Au core dipole resonance. These resonances are shown to be reduced in the Au_rod_Pd_shell_ nanoaprticles (the resonance at ~510 disappearing almost entirely), and completely damped-out by the deposition of Pt, thereby indicating the formation of surface Pt deposition.

**Figure 3:**
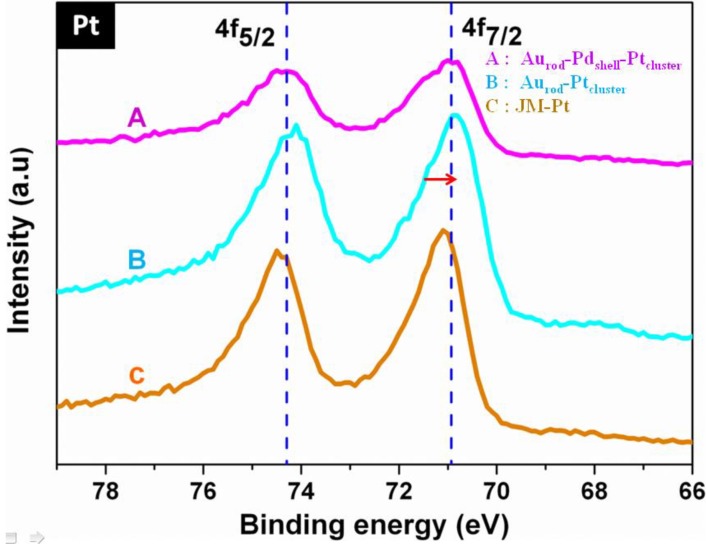
XPS spectra A: Au_rod_-Pd_shell_-Pt_cluster, _B: Au_rod_-Pt_cluster_, C: JM-Pt.

Catalytic efficiency is crucially dependent on the transfer of electron density from the underlying support metal into the surface catalytic architecture, e.g., from Au to Pt in Au_rod_Pt_cluster_, or Au_rod_Pd_shell_Pt_cluster_, see Figure 3. The Pt 4f_5/2_ and 4f_7/2_ peaks for JM-Pt each show, as expected, only one component peak representative of the metal. However the spectra of Au_rod_-Pt_cluster _and especially Au_rod_-Pd_shell_-Pt_cluster_ show peak broadening, possibly due to the presence of multiple components, and a slight shift, due to charge transfer, towards lower binding energies. Similarly the number of unfilled d states (h_Ts_), calculated with respect to a reference Pt foil (defined as 1.6) by a method previously published in the literature [13,14], showed the *d*-band vacancies of Pt for Au_rod_-Pt_cluster_, and the Au_rod_Pd_shell_Pt_cluster_ to decrease to 1.59043 and 1.5501, respectively.

### 3.2. Cyclic Voltammograms (CVs)

**Figure 4:**
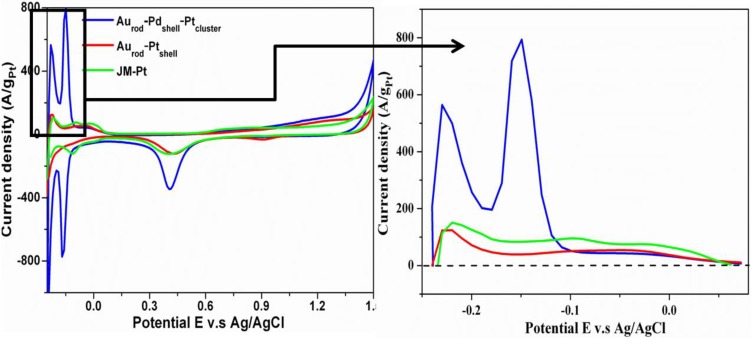
Comparative ECSA data from Au_rod_Pd_shell_-Pt_clusters_, Au_rod_-Pt_shell_ and commercial JM-Pt.

ECSA data derived from the final bimetallic core shell structure that carries the deposited Pt, shown in Figure 4 indicates the significantly higher specific ECSA of the Au_rod_Pd_shell_-Pt_dendritic_ nanostructures when compared to comparative data from Au_rod_-Pt_shell_ and commercial JM-Pt. Figure 4b shows the ECSA profile from −0.25 V to 0.05 V and reflects, in a manner similar to the UV spectra, the transition in structure from a bimetallic shell structure to a Pt dominated surface structure. The calculated comparative hydrogen desorption areas are 55.9, 15.0, and 23.8 AV/g_Pt_ for Au_rod_-Pd_shell_-Pt_cluster_, Au_rod_-Pt_cluster_ and JM-Pt giving respective ECSA values of 1883.0, 1371.7 and 879 cm^2^/mgPt.

Cyclic voltammograms (CVs), made using a Ag/AgCl reference, of Au_rod_/GCE (data not shown), Au-Pd/GCE and Au_rod-_Pd-Pt/GCE, in 0.5 M H_2_SO_4_ solutions at a scan rate of 100 mVs^−1^, revealed that the Au_rod _nanoparticles exhibit a reduction peak at 0.90 V, this peak disappears on coating with Pd and a new reduction peak at approximately 0.38 V appears, while after coating with Pt, an electroactive Pt reduction peak at approximately 0.42 V appeared—allowing us to set the sensing potential to 0.4 V.

### 3.3. H_2_O_2_ Sensing Response

**Figure 5:**
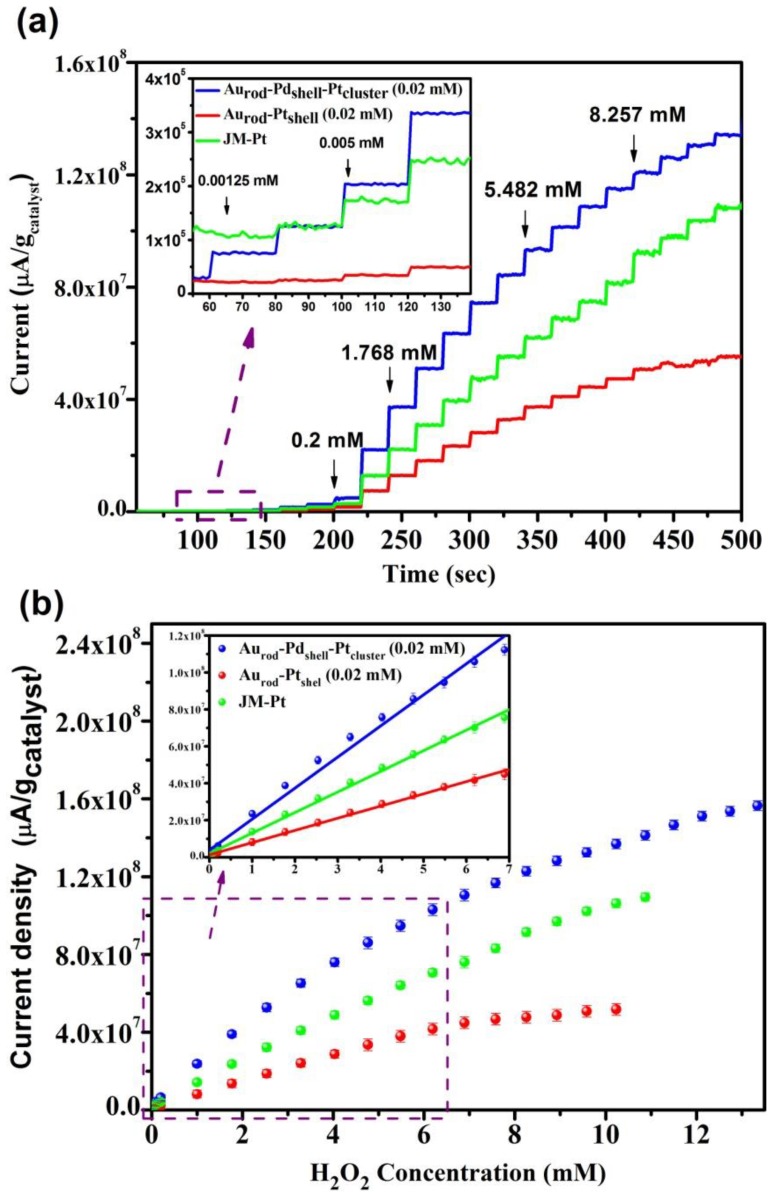
(**a**) shows current density response to the stepwise addition of H_2_O_2_, while (**b**) shows the data plotted as current density verses H_2_O_2 _concentration.

Figure 5a shows the response plotted as current density (µA/gram catalyst) of the Au_rod_-Pd_shell_-Pt_cluster _assembly, together with similar data for AuPt_shell_ and JM-Pt catalysts (data normalized to a constant mass of Pt), to a stepwise challenge by H_2_O_2_; this is re-drawn in Figure 5b to show the response in terms of current density with respect to H_2_O_2_ concentration. The extracted data, tabulated in Table 1, show the sensitivity of the tri-metallic system to be ~2.6 times that of Pt clusters formed as Au_rod_-Pt_cluster_ structures and ~1.6 times that of commercially available JM-Pt.

**Table 1:** Relationship of catalytic structures to hydrogen peroxide sensing.

Applied potential (0.4 V) *vs*. Ag/AgCl	Linear range (µM)	H_2_O_2_ sensitivity (µA/µM×g_cat_)	R^2^
Au_rod_-Pd_shell_-Pt_cluster_	0.00125–6.191	17,721	0.99364
Au_rod_-Pt_cluster_	0.005–5.482	6612	0.99765
JM-Pt	0.005–6.191	11,144	0.9978

Comparison of the measured H_2_O_2 _sensing data with that previously published shows the linear detection range to extend to ultra-low concentrations. The upper detection limit within the linear detection range exceeds that of most recently published studies. In a recent study a linear detection range of 0.012–14 mM was reported [4]; however, the associated sensitivity was 393 μA/mMcm^2^ in contrast with the 604 μA/mMcm^2^ determined in this study.

## 4. Conclusions

The evident synergy resulting from the use of three metals is clearly shown by the significantly higher specific ECSA of the Au_rod_Pd_shell_-Pt_dendritic_ nanostructures when compared to comparative data from Au_rod_-Pt_shell_ and commercial JM-Pt. This is reflected in our H_2_O_2_ sensing response data (which additionally exhibited a slightly extended linear range) that showed the sensitivity of our fabricated mini-sensor, made with the tri-metallic construct, to be 2.68 times greater than an electrode made as a simple Au_rod_-Pt_cluster _and 1.59 times greater than one fabricated with JM-Pt (all under similar conditions).

The core-shell trimetallic catalysts retain a high catalytic ability while using only small amounts of the precious metals, especially palladium and platinum; each sensor being fabricated with a nanoparticle solution (15 μL) comprising a total of 6.7 μg of the active metals (Au 4.9 μg, Pd 1.07 μg and Pt 0.73 μg).

This study, highlighting the use of an optimized tri-metallic rather than a bimetallic structure, serves to highlight the possibility of using tailored combinations of precious catalytic materials, in new morphologies, to address the challenges imposed by the need to make ever more active catalysts using lower amounts of precious materials.
